# Post-translational modification networks in tumor radiosensitivity: mechanistic insights and therapeutic opportunities

**DOI:** 10.1186/s12967-025-07641-6

**Published:** 2026-01-07

**Authors:** Xiaoli Lv, Jiao Xue, Jun Zhou, Qi Zhao, Yandong Liu, Lili Wang, Yang Jiao, Songbing Qin

**Affiliations:** 1 Department of Radiation Oncology, The First Affiliated Hospital of Soochow University, No. 899 Pinghai Road, Suzhou, Jiangsu 215006 China; 2 Department of General Surgery, Affiliated with Suzhou Jiulong Hospital, Shanghai Jiao Tong University, Suzhou, 215006 China; 3 State Key Laboratory of Radiation Medicine and Protection, School of Radiation Medicine and Protection, Key Laboratory of Radiation Damage and Treatment of Jiangsu Provincial Universities and Colleges, Collaborative Innovation Center of Radiological Medicine of Jiangsu Higher Education Institutions, Soochow University, 199 Renai Road, Suzhou, Jiangsu 215123 China

**Keywords:** Post-translational modifications, Tumor radiosensitivity, DNA damage response, Radiosensitization, Therapeutic targets

## Abstract

**Background:**

Radiotherapy is a central modality in cancer management, yet intrinsic and acquired radioresistance and dose-limiting normal tissue toxicities continue to constrain durable tumor control. Beyond genetic and transcriptional programs, post-translational modifications (PTMs) provide rapid and reversible regulation that can rewire radiation responses across tumor and stromal compartments.

**Main body:**

This review synthesizes recent advances defining how major PTM axes, including ADP-ribosylation, ubiquitination and deubiquitination, neddylation, SUMOylation, methylation, acetylation, and lactylation, shape tumor radiosensitivity and radiation-induced injury. We highlight mechanistic links to DNA damage recognition and repair pathway choice, chromatin remodeling, checkpoint control, and cell-fate decisions encompassing apoptosis and ferroptosis. We further discuss how PTM-driven metabolic and redox adaptation influences post-irradiation signaling, and how PTMs modulate anti-tumor immunity by affecting immunogenic cell death, antigen presentation, cytokine networks, and immune checkpoint regulation within the tumor microenvironment. In addition, we summarize emerging evidence for reciprocal crosstalk between PTM enzymes and non-coding RNAs, which can act as upstream regulators, scaffolds, or effectors to reinforce context-specific modification patterns and therapeutic vulnerabilities.

**Conclusions:**

Viewing radioresponse through a PTM-network lens reveals actionable nodes for radiosensitization and radioprotection. Targeting PTM writers, erasers, and readers, alone or in rational combinations with radiotherapy, DNA damage response inhibitors, epigenetic agents, and immunotherapy, may overcome resistance while improving the therapeutic window. Future efforts should prioritize context-specific biomarkers, on-target toxicity management, and mechanism-informed trial designs to translate PTM-guided strategies into clinically meaningful gains.

## Introduction

Cancer continues to impose a staggering global health burden. In 2022, an estimated 20.0 million new cases of cancer were diagnosed worldwide, alongside 9.7 million deaths [1]. Radiotherapy remains a cornerstone of multidisciplinary cancer care. It is recommended that approximately 50% of cancer patients receive radiotherapy, which matches the current global average (rising to 64% when including re-treatment scenarios). This suggests that up to two-thirds of patients may ultimately receive ionizing radiation during their disease course [2]. Despite the development of increasingly precise delivery techniques, intrinsic or acquired radioresistance invariably limits curative outcomes, and normal tissue toxicity persists as a dose-limiting constraint. Conventionally, research endeavors concerning radiation response have focused on genomic alterations and transcriptional programs. However, it is becoming evident that these layers alone cannot fully explain the complexity of tumor radioresponse. A recent paradigm shift has brought PTMs (the dynamic addition or removal of chemical groups on proteins) to the forefront of research as master regulators that operate downstream of gene expression to sculpt cancer cell behavior under radiotherapeutic stress [3].

An extensive array of PTMs, including ADP-ribosylation, ubiquitination, neddylation, SUMOylation, methylation, acetylation, and lactylation, functions as a lexicon of molecular switches. By fine-tuning DNA damage repair processes, orchestrating the balance between cell survival and death (from apoptosis to ferroptosis), modulating oxidative stress responses, and reshaping interactions within the immune tumor microenvironment (TME), PTMs emerge as critical determinants of both tumor radiosensitivity and normal tissue radiotoxicity [4]. Moreover, non-coding RNAs frequently intersect with PTM enzymes, serving as upstream regulators or scaffolds that drive context-specific modification patterns.

**Fig. 1:**
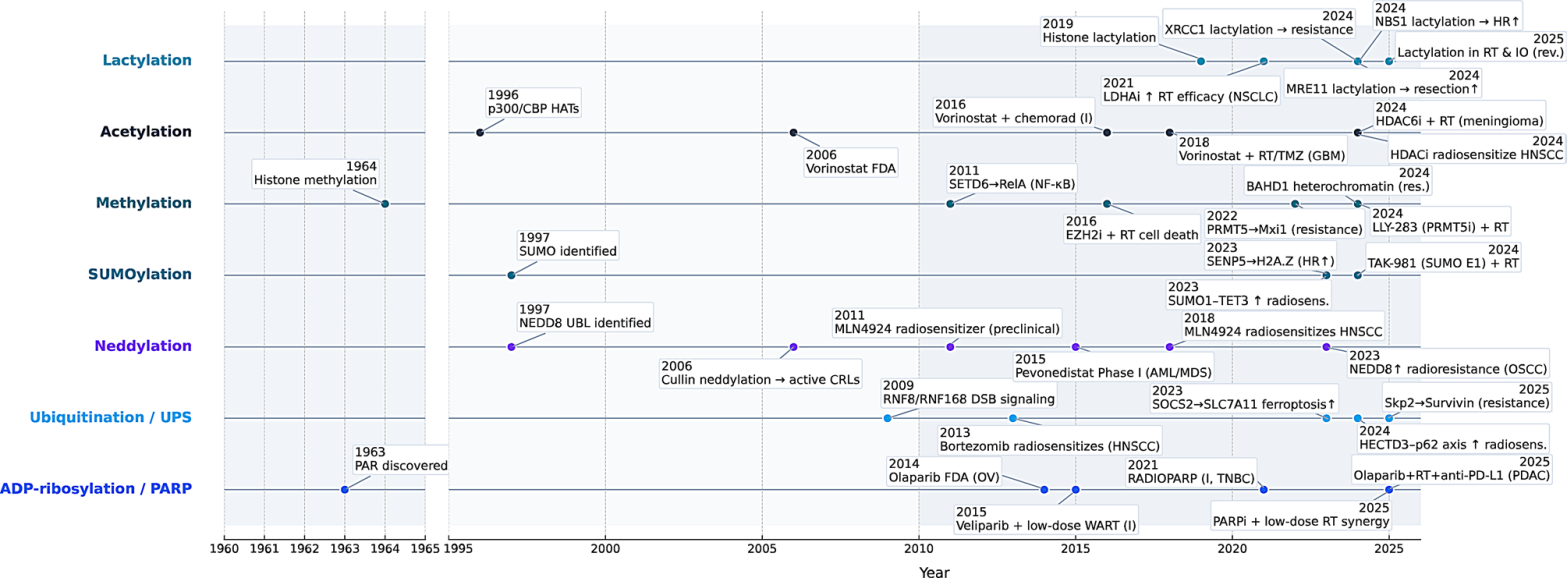
Milestones linking PTM biology to radiotherapy. Timeline summarizing seminal discoveries and translational advances across six post-translational modification (PTM) classes relevant to tumor radioresponse (rows, bottom to top: ADP-ribosylation/PARP, ubiquitination/UPS, SUMOylation, methylation, acetylation, lactylation). Selected events include foundational discoveries (e.g., PAR discovery, SUMO identification), mechanistic inflection points in the DNA-damage response (e.g., RNF8/RNF168 signaling; SENP5–H2A.Z regulation; SUMO1–TET3 stabilization), and clinical or preclinical exemplars of radiosensitization (e.g., FDA approvals and trials of PARP or HDAC inhibitors; PRMT5 inhibitors; TAK-981 for SUMO; LDHA inhibition; combinations such as PARPi + RT ± PD-L1). Markers denote representative—not exhaustive—milestones from 1960–2025 that collectively map how PTM biology has shaped radiotherapy mechanisms and clinical strategies. RT, radiotherapy; PAR/PARP, poly(ADP-ribose)/(poly[ADP-ribose] polymerase); UPS, ubiquitin–proteasome system

In this review, we first survey the mechanisms by which key PTMs influence tumor response to radiation. This includes an integrated discussion of how ADP-ribosylation, ubiquitination/deubiquitination, neddylation, SUMOylation, methylation, acetylation, and lactylation each contribute to DNA damage response (DDR), cell-fate decisions, metabolic reprogramming, and immune modulation in irradiated tumors. We then highlight the crosstalk between PTMs and non-coding RNAs, along with the reciprocal interactions between PTM-mediated signaling and the evolving TME. Finally, emerging therapeutic opportunities exploiting PTM-driven vulnerabilities are discussed, underscoring strategies to overcome radioresistance and enhance the efficacy of radiotherapy in combination with other modalities. Figure 1 presents a schematic, non-scaled timeline aligning seminal PTM discoveries with key mechanistic inflection points in radiobiology and the earliest radiotherapy-relevant translational advances; pharmacologic exemplars are provided as overview markers, with their mechanisms and levels of evidence detailed in subsequent sections.

## Mechanistic landscape of PTM programs in tumor radioresponse

**Fig. 2:**
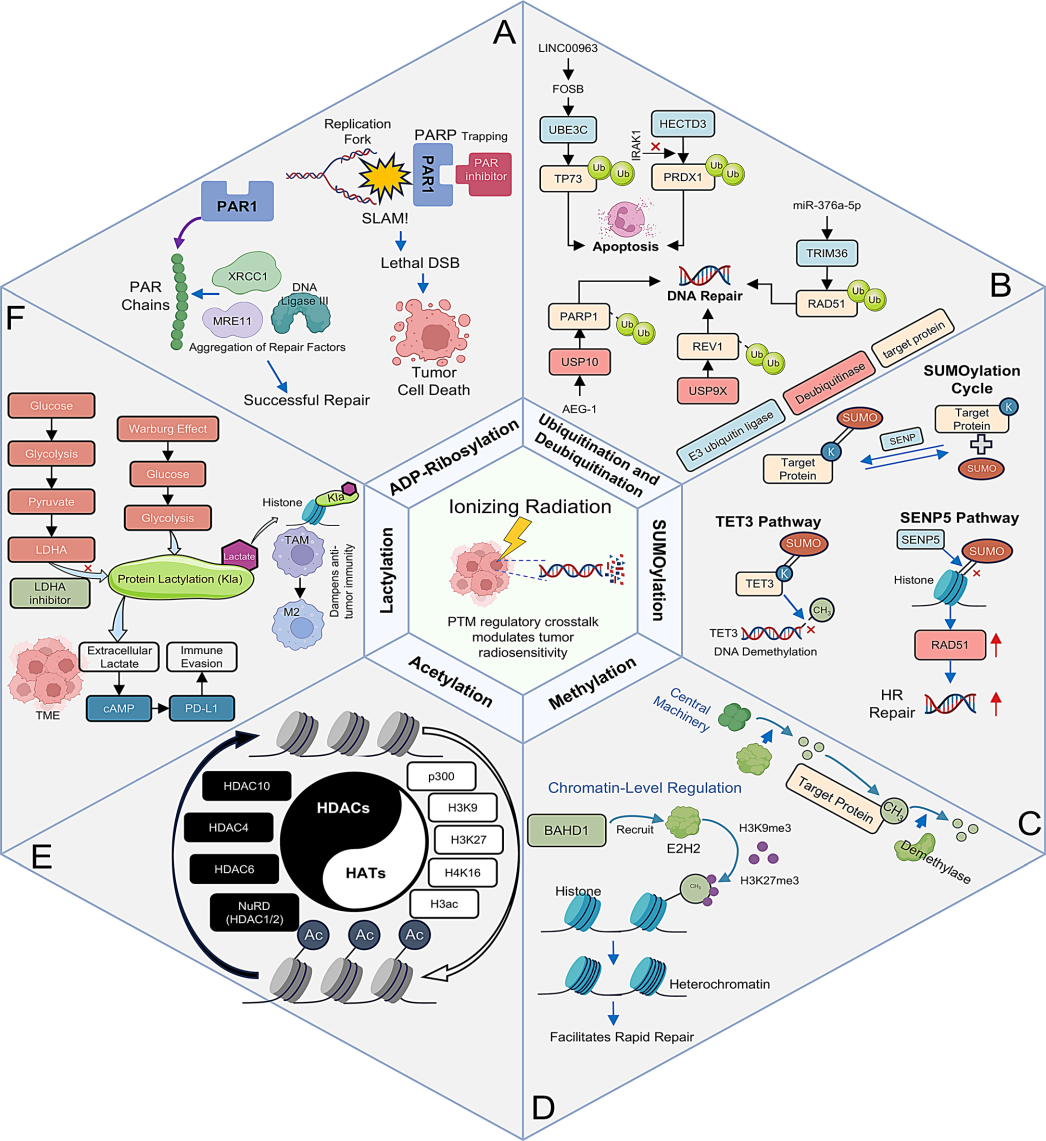
PTM networks coordinating tumor response to ionizing radiation. Schematic overview positioning six PTM axes around ionizing-radiation (IR) stress and illustrating crosstalk between DNA repair, chromatin state, metabolism, and immune signaling. (**A**) ADP-ribosylation: PARP1 senses single-strand breaks and builds PAR chains to recruit XRCC1/MRE11 for repair; PARP inhibitors “trap” PARP on DNA, converting lesions into lethal DSBs. (**B**) Ubiquitination/deubiquitination: E3 ligases (e.g., UBE3C, TRIM36, HECTD3) and DUBs (e.g., USP10, USP9X) tune DNA repair factor stability (REV1/RAD51/PARP1) and apoptosis; RNF8/RNF168-driven histone ubiquitination propagates DSB signaling. (**C**) SUMOylation: reversible SUMO conjugation modulates DDR proteins; SENP5-dependent deSUMOylation of H2A.Z promotes RAD51 loading, whereas SUMO1 modification stabilizes TET3. (**D**) Methylation: BAHD1 recruitment of EZH2 increases H3K9me3/H3K27me3, compacts chromatin and facilitates rapid repair; demethylases counterbalance this. (**E**) Acetylation: the HAT/HDAC axis regulates chromatin accessibility and p53 activity; HDAC overactivity is linked to radioresistance. (**F**) Lactylation: glycolysis/LDHA-derived lactate drives histone and protein lactylation (Kla), influences TAM polarization, and can stabilize DDR factors; LDHA inhibition reduces Kla and may enhance radiosensitivity. DDR, DNA-damage response; DSB, double-strand break; HAT, histone acetyltransferase; HDAC, histone deacetylase; TAM, tumor-associated macrophage; Kla, lysine lactylation. Icons are schematic; not to scale

We next synthesize how radiation-activated PTM programs coordinate DNA damage repair, chromatin remodeling, cell-fate decisions, metabolic rewiring and immune modulation. Figure 2 provides an integrated schema: six PTM axes—ADP-ribosylation/PARP (A), ubiquitination/deubiquitination (B), SUMOylation (C), methylation (D), acetylation (E) and lactylation (F)—are positioned around ionizing-radiation stress, with arrows indicating canonical routes of crosstalk (DDR factors, chromatin state, metabolic/immune interfaces). This framework maps onto Sections 1–6, which elaborate each axis with mechanistic evidence and radiotherapy-relevant consequences.

### ADP-ribosylation: driving DNA repair dynamics and radiosensitization

ADP-ribosylation constitutes one of the earliest signaling events following ionizing radiation, acting as a pivotal regulator at the outset of the DNA damage response. Upon single-strand break (SSB) formation, PARP1 is activated to synthesize long, branched poly(ADP-ribose) (PAR) chains on itself and neighboring chromatin proteins, thereby creating docking sites for scaffold factors such as XRCC1, MRE11, and DNA ligase III [5, 6]. Auto-ADP-ribosylation of PARP1 reduces its chromatin affinity, facilitating transient chromatin relaxation and subsequent factor exchange [7]. Removal of PAR is then mediated by poly (ADP-ribose) glycohydrolase (PARG) and the serine-specific hydrolase ARH3, restoring chromatin compaction and terminating repair signaling [6, 8]. In parallel, the accessory factor HPF1 directs PARP1/2 toward serine residues on histones and other nuclear substrates, generating a Ser-ADPr mark that is specifically reversed by ARH3 to fine-tune repair kinetics [8]. Additionally, mono-ADP-ribosyltransferases such as PARP10 catalyze the attachment of single ADP-ribose units (MARylation) to substrates involved in replication and stress responses. Recent work demonstrates that PARP10 is recruited to nascent strand gaps, orchestrating RAD18-dependent PCNA ubiquitination and translesion synthesis to preserve genome stability under replication stress [9]. This tightly regulated ADP-ribosylation network—comprising PAR synthesis, targeted Ser-ADPr deposition, and coordinated PAR/MAR removal—operates as a master control hub for DNA lesion sensing and repair, and represents a fertile source of targets for radiosensitizing interventions.

#### PARP1-dependent repair pathways and radiosensitivity

Cells or organisms that lack PARP1 show extreme sensitivity to ionizing radiation – they accumulate DNA damage and undergo apoptosis due to their inability to repair single-strand breaks (which convert to lethal double-strand breaks) [10]. Conversely, high PARP1 activity can protect tumor cells from radiation by swiftly repairing DNA breaks. In 2023, Li et al. provided a comprehensive review of PARP-1’s multifaceted role in radioprotection and radiotherapy [11].

Preclinically, PARP inhibitors (PARPi) have demonstrated potent radiosensitizing effects across diverse tumor models. In glioblastoma stem-like cells (GSCs), Paul Lesueur et al. showed that talazoparib (10 nM) combined with photon (4 Gy) or carbon‐ion irradiation dramatically reduced GSC fraction in R633 and TG1 lines, induced a marked and prolonged G₂/M block, impaired proliferation and lowered clonogenic survival [12]. Similarly, in head-and-neck squamous cell carcinoma (HNSCC), Mentzel et al. reported that talazoparib (50 nM) and niraparib (2.5 µM) each significantly enhanced IR-induced apoptosis and decreased colony formation in both HPV-positive (UD-SCC-2, UM-SCC-47) and HPV-negative (Cal33, CLS-354, Detroit 562, HSC4, RPMI2650) cell lines—while sparing normal fibroblasts from radiosensitization [13]. These studies establish PARPi as robust enhancers of radiation-induced DNA damage and mitotic catastrophe in tumor cells, supporting their integration into radiotherapy regimens.

Clinically, the single-institution RadioPARP phase I trial (NCT03109080) demonstrated that olaparib at escalating doses (50–200 mg BID), initiated one week before and continued throughout breast‐conserving radiotherapy in 24 high‐risk Triple-negative breast cancer (TNBC) patients, was well tolerated with no dose‐limiting toxicities observed. No grade ≥ 3 late effects were seen at two-year follow-up [14]. In a recent review, the authors critically assessed the translational readiness of combining PARP inhibitors with radiotherapy, highlighting extensive preclinical evidence of enhanced tumor radiosensitivity and advocating for imminent clinical integration [15].

#### Noncanonical PARPs in modulating tumor radioresponse

Beyond PARP1, emerging evidence highlights additional PARP family members as key modulators of tumor radioresponse. For example, PARP7 (ARTD14) functions as a nuclear mono-ADP-ribosyltransferase. It MARylates the AP-1 subunit FRA1 at C97, shielding FRA1 from PSMC3-mediated proteasomal degradation. Stabilized FRA1 represses IRF1/IRF3-dependent apoptotic and interferon-stimulated genes, fostering an immune-evasive, apoptosis-resistant state—pharmacologic inhibition of PARP7 with RBN-2397 or mutation of FRA1 C97 abrogates MARylation, promotes FRA1 turnover, unleashes IRF1/IRF3 signaling and CASP8-driven apoptosis, and sensitizes lung and breast cancer cells to genotoxic stress [16]. PARP7 also MARylates the aryl hydrocarbon receptor (AHR) and ERα, marking them for ubiquitin-mediated degradation and thereby modulating oxidative-stress and hormone-driven pathways that intersect with DNA repair [17].

Similarly, PARP3 is rapidly recruited to DNA double-strand breaks, where it engages core non-homologous end-joining factors (Ku70/Ku80, DNA-PKcs and XRCC4–Ligase IV), catalyzing their PARylation to accelerate classical Non-homologous end joining (NHEJ). Genetic depletion of PARP3 delays γH2AX foci resolution, impairs DSB repair kinetics and increases radiosensitivity in human cells and mouse models [18].These insights extend ADP-ribosylation’s role in radiobiology beyond PARP1, revealing PARP7 and PARP3 as promising targets for radiosensitization strategies that combine DNA repair inhibition with modulation of anti-tumor immunity.

In line with these findings, the therapeutic landscape is expanding to target multiple nodes within the ADP-ribosylation pathway beyond PARP1. Inhibitors of PARG, the primary enzyme responsible for PAR catabolism, have emerged as a conceptually distinct class of radiosensitizers that prevent the turnover of DNA damage signals and prolong PAR-dependent DDR signaling [19, 20]. By integrating PARP-targeting agents with radiotherapy, and in some settings leveraging the immunomodulatory functions of PARP7 to boost type I IFN signaling and anti-tumor immunity, it is possible to shift the DNA repair equilibrium toward tumor cell lethality while aiming to spare normal tissues [11, 17]. Tumor-type differences in ADP-ribosylation dependence are increasingly recognized: in glioblastoma, constitutive PARP1 activation in glioma-initiating cells contributes to an intrinsically radioresistant phenotype [21], whereas in BRCA-deficient breast cancers, particularly TNBC, reliance on PARP-mediated repair creates a synthetic-lethal vulnerability that PARP inhibitors exploit and that can be combined with radiotherapy to selectively enhance radiosensitivity [22, 23] (Fig. 2A).

### Ubiquitination and deubiquitination: tipping the balance of radiosensitivity

Building on PARP-driven DDR signaling, ubiquitin-mediated proteolysis constitutes a critical post-irradiation regulatory layer that dictates cell fate. Ubiquitination proceeds via an ATP-dependent E1–E2–E3 cascade. The type of ubiquitin chain attached (e.g. K11/K48 linkages versus K63/M1 or other linkages) determines substrate fate, with K11/K48 chains targeting proteins for proteasomal degradation and K63/M1 chains mediating scaffolding or signaling functions [24]. Upon DSB formation, RNF8 catalyzes K63-linked ubiquitination of histone H2A. RNF168 then amplifies this signal by catalyzing additional K63 ubiquitination on H2A at lysines 13 and 15, recruiting BRCA1 and 53BP1 to orchestrate DSB repair [25]. Recent work reveals that the ubiquitin reader ZNF451 collaborates with RNF8 to spatially regulate RNF168 localization and amplify K63-linked chains, thereby sustaining DDR signaling and fostering radioresistance [26]. Notably, depletion of ZNF451 sensitizes tumor cells to IR-induced apoptosis. Moreover, the E3 ligase HECTD3 promotes K63-linked ubiquitination of p62, driving autophagy-dependent chromatin remodeling that limits RNF168 recruitment; pharmacologic inhibition of the HECTD3–p62 axis enhances radiosensitivity in TNBC models [27]. Conversely, the F-box E3 ligase Skp2 assembles mixed K48/K63 ubiquitin chains on Survivin, stabilizing this apoptosis inhibitor and conferring radioresistance in oral squamous cell carcinoma [28]. In parallel, deubiquitinases fine-tune this balance: USP9X removes ubiquitin from the TLS polymerase REV1, preserving its function in HR-promoting Rad18 recruitment and driving lung cancer radioresistance [29]. Broad-spectrum DUB inhibitors (e.g., PR-619) have been shown to impair DDR factor recruitment, amplify IR-induced DNA damage signaling, and sensitize tumors to radiotherapy in preclinical studies [30]. This dynamic interplay between E3 ligases and DUBs precisely calibrates repair versus death decisions following irradiation, positioning the ubiquitin–proteasome system as a pivotal determinant of radiosensitivity.

#### E3 ubiquitin ligases in radioresistance

Emerging evidence highlights that specific E3 ubiquitin ligases selectively modulate DNA damage repair and apoptotic pathways, thereby dictating tumor radiosensitivity. In the context of breast cancer, the long non-coding RNA (lncRNA) LINC00963 has been observed to facilitate the recruitment of FOSB to the UBE3C promoter. This process results in the upregulation of UBE3C, leading to the K48-linked ubiquitination and proteasomal degradation of the pro-apoptotic TP73 protein. Consequently, this mechanism serves to impede radiation-induced apoptosis and promote radioresistance [31]. In lung adenocarcinoma, the RING-type E3 ligase TRIM36 binds RAD51, facilitating its ubiquitination and subsequent degradation. This axis is subject to negative regulation by microRNA-376a-5p, and the restoration of TRIM36 expression has been shown to significantly enhance radiosensitivity by impairing homologous recombination (HR) repair [32]. At the sites of DNA double-strand breaks, RNF168 has been shown to mediate K63-linked ubiquitination of histone H2A/H2AX, thus recruiting 53BP1 and propagating the DNA damage response. Conversely, glioma stem-like cells overexpressing G0S2 have been observed to suppress RNF168 via mTOR-S6K signaling, resulting in reduced 53BP1 focus formation and subsequent sensitization of tumors to radiotherapy. Notably, these effects have been demonstrated to be reversible upon restoration of RNF168 [33]. Finally, in glioblastoma, radiation-induced O-GlcNAcylation of MLPH at Ser510 impedes its recognition by the E3 ligase TRIM21, leading to MLPH stabilization, NF-κB activation, and enhanced radioresistance. Inhibition of MLPH or the restoration of TRIM21-mediated ubiquitination has been shown to restore radiosensitivity [34]. Collectively, these studies illustrate how E3 ligases integrate chromatin modification, non-coding RNA regulation and stress signaling to orchestrate tumor radioresponse and reveal multiple targets for radiosensitization.

#### Ubiquitin-mediated cell-death and survival signaling in radiosensitivity

Ubiquitination also governs regulated cell-death pathways that determine tumor radioresponse. In hepatocellular carcinoma (HCC), SOCS2 serves as an E3-adaptor. Following irradiation, the SH2 domain of SOCS2 recognizes the N-terminus of SLC7A11, and its SOCS-box recruits elongin B/C and ubiquitin. This process promotes K48-linked polyubiquitination and proteasomal degradation of SLC7A11. As a result, cystine import is depleted, lipid peroxidation and Fe²⁺ accumulation are elevated, and ferroptosis and radiosensitization are triggered [35].

Conversely, some ubiquitin pathways evidently confer radioresistance by stabilizing pro-survival factors. Survivin (BIRC5), an inhibitor of apoptosis, was reported to be stabilized by the E3 ligase Skp2 in radioresistant oral squamous carcinoma, thereby linking the overactive ubiquitin ligase to impaired apoptotic response after irradiation [28]. Concurrently, recent research in gliomas has revealed that ionizing radiation can itself activate an oncogenic ubiquitin-mediated cascade. Similarly, TRAF4 interacts with Akt under IR, catalyzing its K63-linked ubiquitination and thereby maintaining Akt signaling. This process, in turn, leads to the phosphorylation of MCL-1 at S159, which results in the obstruction of JOSD1-mediated deubiquitination. The outcome of this process is the stabilization of MCL-1 and the inhibition of mitochondrial apoptosis. Genetic or pharmacologic targeting of TRAF4/MCL-1 has been demonstrated to reinstate radiosensitivity in OSCC models [36].

IRAK, a kinase involved in immune signaling, was found to be upregulated by radiation through a cGAS-STING-dependent transcription pathway. Additionally, it was observed to bind directly to the antioxidant enzyme PRDX1. IRAK1 prevents PRDX1 from being ubiquitinated and subsequently degraded by the E3 ligase HECTD3. The result of this process is the accumulation of PRDX1, which functions as an antioxidant and has been shown to inhibit radiation-induced autophagy, a process that can lead to cell death. This effect, therefore, contributes to the protection of glioma cells from potential lethal damage. The study demonstrated that the inhibition of IRAK1 or the restoration of PRDX1 ubiquitination re-sensitized glioma cells to radiation by facilitating ROS-mediated autophagy [37]. This *IRAK1–PRDX1 axis* demonstrates the capacity of a stress-inducible E3-interfering factor to facilitate tumor cell survival under radiotherapy.

#### Deubiquitinases as gatekeepers of DNA repair and determinants of radioresistance

Recent findings have identified deubiquitinases (DUBs), which are counterparts to E3 ligases, as pivotal “gatekeepers” of the DDR. These DUBs have been shown to play a key role in coordinating various signaling pathways, including double-strand break (DSB) signaling, cell-cycle checkpoints, and apoptosis. Additionally, DUBs have been observed to regulate hypoxia responses, thereby modulating tumor radiosensitivity. A comprehensive review by Cao et al. detailed these multifaceted roles and highlighted DUBs as promising radiosensitization targets [38]. For example, USP9X was recently shown to deubiquitinate the translesion synthesis polymerase REV1, thereby preventing its degradation in irradiated lung cancer cells. Stabilized REV1 promotes tolerance to DNA damage. In addition, the knockdown of USP9X has been shown to increase radiosensitivity by tipping the balance toward lethal DNA damage accumulation [29]. In esophageal squamous cell carcinoma, the astrocyte elevated gene-1 (AEG-1) has been shown to recruit USP10, resulting in the deubiquitination of PARP1. This process has been demonstrated to enhance homologous recombination–mediated DSB repair and to confer radioresistance. Conversely, the disruption of USP10 or AEG-1 has been observed to render tumors more susceptible to radiation-induced DNA damage [39]. Moreover, genetic depletion or IU1-mediated pharmacologic inhibition of USP14 impairs both non-homologous end joining and homologous recombination in non-small cell lung cancer (NSCLC), markedly reducing clonogenic survival post-irradiation [40]. A recent study also identified USP28 as a driver of HCC radioresistance by deubiquitinating and stabilizing WDHD1, thereby promoting tumor progression and IR tolerance. Conversely, USP28 silencing enhanced radiosensitivity in HCC xenografts [41]. These findings illustrate how DUBs can influence the balance between repair and death in irradiated tumors, and underscore the therapeutic promise of small-molecule DUB inhibitors (e.g., IU1) and emerging deubiquitinase-targeting chimeras (DUBTACs) to dismantle resistance circuits and potentiate radiotherapy [38].

Collectively, the ubiquitin-proteasome system emerges as a bifurcated regulator of tumor radioresponse, orchestrating protein turnover to either amplify cell death or fortify resistance. By precisely calibrating the stability of key effectors - ranging from DNA repair machinery to apoptosis mediators-E3 ligases and DUBs establish a dynamic network that determines cellular fate upon irradiation. The recent delineation of pivotal nodes, including UBE3C, IRAK1, Skp2, USP9X and USP10, unveils tractable targets for the development of context-specific radiosensitizers. It is important to note that ubiquitination circuits are deeply integrated with signaling cascades and non-coding RNA regulators, thereby underscoring the multilayered architecture of radiosensitivity control. Therapeutic strategies that target the ubiquitin-proteasome axis in conjunction with radiotherapy demonstrate considerable potential in leveraging these interdependencies and overcoming resistant phenotypes, as evidenced by emerging preclinical models. Importantly, different tumors rely on distinct ubiquitin pathways to modulate radioresponse. In breast cancer, LINC00963 promotes nuclear translocation of FOSB and transcriptional upregulation of UBE3C, which induces ubiquitin-dependent degradation of TP73 and confers radioresistance [31]. In lung adenocarcinoma, reduced TRIM36 expression is associated with elevated RAD51 levels and radioresistance, whereas restoring TRIM36 or relieving repression by miR-376a-5p enhances RAD51 ubiquitination and markedly increases radiosensitivity [32]. Together, these examples illustrate how ubiquitin-mediated DDR and cell-death mechanisms vary across tumor types and contribute to heterogeneous radiation responses (Fig. 2B).

### Neddylation: linking cullin–RING ligases and ubiquitin signaling in radiosensitivity

Neddylation is a ubiquitin-like post-translational modification in which the small protein NEDD8 is covalently attached to target substrates, most prominently the cullin scaffold subunits of cullin-RING E3 ubiquitin ligases (CRLs). This modification functions as a master regulatory switch for CRLs: in an ATP-dependent cascade analogous to ubiquitylation, NEDD8 is activated by the NEDD8-activating enzyme NAE1/UBA3, transferred to the E2 conjugating enzyme UBE2M (also known as Ubc12) or UBE2F, and finally ligated to conserved lysine residues on cullin proteins by RING-type E3 ligases such as RBX1 and RBX2 [42, 43]. Neddylation induces characteristic conformational rearrangements in cullins that relieve CAND1-mediated inhibition and markedly enhance CRL ubiquitin ligase activity [44]. Through this mechanism, CRLs promote timely ubiquitylation and proteasomal degradation of many cell-cycle regulators, replication licensing factors and DNA damage response proteins, thereby dynamically controlling processes that are central to how tumor cells respond to ionizing radiation [42, 45]. This pathway is counterbalanced by deneddylation: the COP9 signalosome (CSN) removes NEDD8 from cullins, returning CRLs to an inactive state and fine-tuning ubiquitin signaling output [45, 46]. Dysregulation of this neddylation–deneddylation cycle can therefore influence radiosensitivity. Hyperactivated neddylation accelerates the turnover of proteins that restrain cell proliferation or promote senescence, whereas impaired neddylation causes accumulation of CRL substrates that may either favor cell death or, when coupled to defective checkpoint control, drive genomic instability and radioresistance [43].

Pharmacologic blockade of the neddylation pathway has emerged as a promising strategy to enhance tumor radiosensitivity. The small-molecule MLN4924 (pevonedistat) is a first-in-class, selective NEDD8-activating enzyme (NAE) inhibitor that functionally inactivates CRLs and triggers the accumulation of their normally short-lived substrates [42, 47]. Upon MLN4924 treatment, cancer cells fail to degrade key negative regulators of cell-cycle progression and DNA replication, including the checkpoint kinase WEE1, the CDK inhibitors p21^(Cip1)^ and p27^(Kip1)^, and the replication licensing factor CDT1, leading to catastrophic rereplication stress and checkpoint activation [48, 49]. Under irradiation, this substrate accumulation translates into an amplified DNA damage burden, sustained G2/M checkpoint arrest, and heightened apoptosis in tumor cells [48, 49]. Notably, MLN4924 selectively radiosensitizes tumor cells while relatively sparing normal tissues, as shown in pancreatic cancer models in which MLN4924 increased IR-induced DNA double-strand breaks and apoptosis through CDT1- and WEE1-dependent mechanisms without exacerbating normal tissue toxicity [49]. MLN4924-mediated CDT1 stabilization similarly drives replication stress and mitotic catastrophe in head-and-neck squamous cell carcinoma (HNSCC) [50]. In breast cancer, neddylation inhibition enhances radiotherapy efficacy through a predominantly p21-dependent G2 arrest [51]. In castration-resistant prostate cancer, accumulation of WEE1, p21, and p27 underlies a profound G2/M blockade and apoptotic cell death after IR [52]. Collectively, these findings underscore that different tumor types exploit distinct CRL substrate circuits, such as CDT1-driven rereplication stress and p21-dominated cell-cycle arrest, which act as context-specific Achilles’ heels that can be targeted by neddylation inhibition to overcome radioresistance.

Beyond cell-intrinsic effects, neddylation intersects with the ubiquitin system to regulate DNA repair pathways that are critical for the radioresponse. Neddylation and subsequent deneddylation of cullin–RING ligases fine-tune the stability and activity of multiple DNA-damage-response proteins, including core components of non-homologous end joining (NHEJ) [53]. A representative example is the CUL1–FBXW7 CRL1 ligase, which catalyzes K63-linked polyubiquitylation of XRCC4 on Lys296 after irradiation; this non-degradative ubiquitin signal enhances XRCC4 association with Ku70/Ku80 and promotes efficient NHEJ repair. Genetic depletion of FBXW7 or pharmacologic attenuation of neddylation reduces XRCC4 ubiquitylation, impairs NHEJ, increases persistence of radiation-induced double-strand breaks and sensitizes tumor cells to ionizing radiation [54]. In addition to DNA repair, neddylation also shapes tumor–immune crosstalk. In glioblastoma, inhibition of the NEDD8-activating enzyme by pevonedistat stabilizes c-MYC, upregulates PD-L1 expression and enhances the therapeutic benefit of anti-PD-L1 antibodies [55]. A complementary study showed that MLN4924 induces PD-L1 via the MEK–JNK–AP-1 axis, thereby driving cancer-associated immunosuppression that can be reversed by combining MLN4924 with MEK inhibition or PD-L1 blockade [56]. Together with broader work linking neddylation to innate and adaptive immune responses, these data place the NEDD8 pathway at a central node connecting ubiquitin-dependent proteolysis, DNA repair signaling and anti-tumor immunity. Clinically, the first-in-class NAE inhibitor pevonedistat has already been evaluated in phase I trials in patients with acute myeloid leukemia and myelodysplastic syndromes, demonstrating on-target pathway blockade, manageable toxicity and preliminary antitumor activity [57]. Subsequent clinical studies and recent comprehensive reviews underscore the continued development of pevonedistat-based combination regimens across a range of solid and hematologic malignancies [58]. Collectively, these findings support neddylation inhibition as a rational strategy to disrupt ubiquitin-mediated DNA repair signaling, remodel the tumor immune microenvironment and sensitize cancers to radiotherapy.

### SUMOylation: modulating the DNA damage response and tumor radiosensitivity

In parallel with ubiquitin-mediated signaling, SUMOylation—catalyzed via SENP protease processing of SUMO precursors, activation by the SAE1/UBA2 E1 heterodimer, transfer by the sole E2 enzyme Ubc9, and substrate ligation by PIAS-family E3s—constitutes a pivotal PTM cascade that orchestrates the DNA damage response [59, 60]. Unlike ubiquitin, SUMO conjugation does not target proteins for degradation but instead modulates protein–protein interactions, subcellular localization, and enzymatic activities, with SENP family proteases effecting rapid deSUMOylation to reset signaling [59]. Upon ionizing radiation, key DNA repair factors are dynamically SUMOylated to nucleate repair assemblies: PIAS4-mediated SUMOylation of MDC1 promotes its recognition by the STUbL RNF4 and subsequent chromatin remodeling [59]; SUMO2/3 modifications of the BRCA1–BARD1 heterodimer are precisely removed by SENP6 to regulate their timely recruitment to double-strand breaks [61]; and TOPORS-dependent SUMOylation of RAD51 at Lys57/Lys70 enhances RAD51 chromatin loading and homologous recombination efficiency [62]. Emerging evidence also highlights novel SUMO-dependent regulators: the SUMO reader ZNF451 catalyzes SUMO2 conjugation of RNF168, stabilizing its accumulation at damage sites and intensifying repair foci formation [26]. Misregulation of any node within this SUMO network perturbs repair kinetics and cell‐fate decisions, positioning the SUMO pathway as a crucial determinant of tumor radiosensitivity.

#### Stabilizing pro-radiation factors via SUMO1

Recent evidence indicates that SUMO1-mediated modification of the DNA demethylase TET3 is essential for its ability to enhance radiosensitivity in colorectal cancer. TET3, a 5-methylcytosine dioxygenase, can activate expression of tumor-suppressive genes by demethylating their promoters. Liu et al. found that higher TET3 levels correlated with greater radiosensitivity in colorectal cancer cells and patient samples. Overexpressing TET3 led to more radiation-induced DNA damage, G2/M arrest, and apoptosis, confirming TET3’s pro-sensitivity role. The twist came when they discovered TET3 is modified by SUMO1 (and SUMO2/3) at multiple lysine sites, and this SUMOylation markedly increases TET3 protein stability. SUMOylation did not change TET3’s nuclear localization, but it protected TET3 from degradation, thus sustaining its DNA demethylation activity in the nucleus. If TET3 was not sumoylated (for instance, mutating SUMO attachment sites led to faster turnover), the cells became more radioresistant [63]. These results identify SUMO1-mediated stabilization of TET3 as a novel mechanism to maintain an epigenetic state favorable for radiotherapy response. It also links DNA demethylation (an epigenetic mark) with a post-translational mark (SUMO) – bridging two layers of regulation. Clinically, it suggests that tumors with deficiencies in the SUMO conjugation pathway might exhibit radioresistance due to failure to sustain TET3 or similar factors after irradiation.

#### SENP proteases and DNA repair

SUMOylation is reversed by sentrin/SUMO-specific proteases (SENPs), whose dysregulation can reprogram tumor radiosensitivity. A landmark study by Liu et al. identified SENP5 as a driver of radioresistance in colorectal cancer. High SENP5 levels in patient biopsies correlated with poor neoadjuvant‐RT response, while CRISPR/Cas9‐mediated SENP5 knockout in CRC cell lines and xenografts markedly enhanced IR‐induced cell killing. Mechanistically, SENP5 promotes homologous recombination by deSUMOylating the histone variant H2A.Z at DNA‐break sites, enabling efficient RAD51 foci formation and DSB repair; loss of SENP5 leads to persistent H2A.Z SUMOylation, defective RAD51 recruitment and accumulation of unrepaired breaks [64]. These findings nominate the SENP5–H2A.Z axis as both a predictive biomarker of RT response and a therapeutic vulnerability: pharmacologic or genetic inhibition of SENP5 deSUMOylation activity offers a promising strategy to dismantle high‐fidelity repair and sensitize tumors to radiotherapy.

#### SUMO interplay with other PTMs

SUMOylation dynamically intersects with ubiquitination and phosphorylation to calibrate the DNA-damage response. For example, PIAS1‐mediated SUMOylation of MRE11 at DSB sites shields it from RNF4‐dependent ubiquitination and proteasomal degradation, preserving MRE11’s exonuclease activity and enabling efficient DNA end resection; subsequent deSUMOylation by SENP3 restores the balance, illustrating a finely tuned SUMO–ubiquitin crosstalk that governs repair kinetics [65]. Similarly, SUMO1 conjugation of the tumor suppressor p53 at Lys386 by PIAS-family E3 ligases and TOPORS is essential for full transcriptional activation of pro‐apoptotic target genes in response to IR; loss of this modification diverts p53 toward cell‐cycle arrest programs and attenuates apoptosis, thereby promoting radioresistance [66].Beyond canonical DDR factors, SUMOylation of metabolic and epitranscriptomic regulators also influences radioresponse. In colorectal cancer, SUMO1‐mediated modification of the m⁶A “writer” METTL3 at Lys459 enhances its protein stability and drives oncogenic circRNA production; METTL3‐K459R mutants exhibit reduced tumor growth and heightened radiosensitivity, revealing a SUMO–circRNA axis as a novel therapeutic target [67].In summary, dynamic SUMO conjugation shapes tumor radioresponse by modulating the fate of discrete effectors. SUMO1-mediated stabilization of TET3 sustains pro-death epigenetic programs in colorectal cancer, whereas SENP5‐driven deSUMOylation of H2A.Z preserves high-fidelity homologous recombination and underlies radioresistance. This substrate‐specific “SUMO code” presents both a challenge and an opportunity: global blockade of SUMO conjugation through E1 inhibitors could broadly sensitize diverse tumors, while precision targeting of individual SENPs—for example, SENP5 antagonists in HR-addicted cancers—offers a tailored radiosensitization strategy. As the post-irradiation SUMO proteome is mapped in greater detail, these insights will guide the development of next-generation radiosensitizers that exploit the nuanced interplay between SUMOylation and genome stability (Fig. 2C).

Notably, the influence of SUMO pathways on radiation response is heterogeneous across cancers. In colorectal cancer, SENP5 overexpression removes SUMO marks from repair proteins such as H2A.Z, enhances HR efficiency and confers radioresistance [64]. In the same setting, robust SUMO1 conjugation of TET3 stabilizes the protein, promotes radiation-induced apoptosis and is associated with increased radiosensitivity [63] In p53-dependent tumors, including subsets of breast cancer, SUMOylation of p53 enhances its transcriptional activity toward pro-apoptotic targets, whereas loss of this modification or increased deSUMOylase activity weakens p53-mediated apoptosis after IR and favors transient cell-cycle arrest and survival [68, 69]. Together, these examples highlight how SUMO-regulated DNA repair and death programs are wired differently across tumor types, contributing to heterogeneous radiation outcomes.

### Methylation: coordinating DNA repair and chromatin in radioresponse

Beyond ubiquitin-like conjugations, covalent methylation of proteins provides an additional epigenetic and signaling layer that shapes the radiation response. Protein methylation is enacted by S-adenosylmethionine (SAM)-dependent methyltransferases, including SET-domain lysine methyltransferases (e.g., SET7, SUV39H1, EZH2) and protein arginine methyltransferases (PRMT1, PRMT5), which deposit mono-, di- or tri-methyl marks on histone and non-histone substrates [70, 71]. These marks regulate chromatin accessibility, transcriptional programs, and the recruitment or stability of DNA repair factors—H3K4me3 correlates with active chromatin, whereas H3K9me3 and H3K27me3 enforce heterochromatin and can modulate double-strand break (DSB) repair dynamics. Indeed, elevated H3K27me3 contributes to radioresistance in nasopharyngeal carcinoma, and pharmacologic inhibition with GSK126 restores apoptosis by derepressing pro-death genes [72]. Removal of methyl marks is achieved by lysine demethylases such as KDM1A/LSD1 and Jumonji C domain enzymes, ensuring dynamic control of methylation states during the DNA damage response. LSD1 is rapidly recruited to DSB sites, where it demethylates H3K4me2 to facilitate repair complex assembly and checkpoint activation [73]. Together, these opposing enzyme classes establish a finely tuned “methylation code” that dictates chromatin structure, DNA repair kinetics, and ultimately tumor radiosensitivity. Mapping this post-irradiation methylome promises to uncover reversible targets for next-generation radiosensitizers.

#### Heterochromatin and radioresistance

A recent multi-omics study of prostate and head/neck cancer models rendered radioresistant by fractionated IR identified the BAH domain–containing protein BAHD1 as a key driver of enhanced heterochromatin formation. BAHD1 overexpression coincided with global increases in H3K9me3 and H3K27me3; conversely, siRNA-mediated BAHD1 knockdown reduced these repressive marks, delayed γH2AX foci clearance, impaired DNA-PK–dependent end-joining and fully reversed the radioresistant phenotype in clonogenic assays [74]. Clinically, high BAHD1-driven heterochromatin enrichment scores were associated with significantly higher local relapse rates after radiotherapy [74]. These findings implicate BAHD1-dependent heterochromatin modulation as causal in acquired radioresistance, and suggest that disrupting this axis—either by inhibiting heterochromatin “writers” (e.g., EZH2) or blocking BAHD1 recruitment—could re-sensitize tumors to IR.

Preclinical studies corroborate the radiosensitizing potential of targeting heterochromatin: the EZH2 inhibitor GSK126 downregulates EZH2 and sensitizes castration-resistant prostate cancer cells to IR- and CPT-induced cell death in xenografts [75]. Furthermore, depletion of EZH2 in glioma stem-like cells abolishes their PRC2-dependent radioresistance and enhances IR-induced apoptosis [75]. These data establish heterochromatin and its regulators as tractable targets for radiosensitization.

#### PRMT-driven arginine methylation in tumor radiosensitivity

Arginine methyltransferases (PRMTs) orchestrate critical pro-survival pathways in the IR response by modifying non‐histone substrates. In non–small cell lung cancer (NSCLC), PRMT5 symmetrically dimethylates the transcriptional repressor Mxi1, creating a phosphodegron that recruits the β‐TrCP E3 ligase to ubiquitinate and degrade Mxi1. Genetic silencing of PRMT5 impairs DSB repair (as evidenced by persistent γH2AX foci) and heightens radiosensitivity in vitro and in xenografts; these effects are partially reversed by co-silencing Mxi1, pinpointing Mxi1 as a key downstream effector. Pharmacologic inhibition with the selective PRMT5 inhibitor EPZ015666 produces “extraordinary” radiosensitization, with treated tumors exhibiting significantly reduced DNA repair and increased IR‐induced cell death [76].

Broader applicability was demonstrated using LLY-283, another PRMT5 inhibitor: treatment of U251 glioma, PSN1 pancreatic and MDA-MB-231 breast cancer cells with LLY-283 (100 nM) markedly decreased PRMT5 activity, inhibited DSB repair, altered RNA splicing of DDR genes, and enhanced IR-induced clonogenic kill—while sparing MRC9 fibroblasts. In U251 xenografts, LLY-283 (100 mg/kg) administered prior to fractionated IR significantly delayed tumor growth, confirming PRMT5 as a tumor‐selective radiosensitization target [71].

Upstream activation of PRMT5 by stanniocalcin 2 (STC2) has been reported in esophageal squamous cell carcinoma: STC2 enhances PRMT5 activity to boost DSB repair and inhibit ferroptosis, thereby promoting radioresistance; genetic or pharmacologic blockade of PRMT5 abrogates this effect and restores IR sensitivity [77]. In parallel, PRMT1-dependent methylation of BRCA1 is induced by IR in breast cancer cells. PRMT1 silencing shifts BRCA1 from a nuclear repair‐promoting role toward cytoplasmic pro‐death signaling, impairs homologous recombination and sensitizes cells to IR [78]. Collectively, these studies position PRMT5 and PRMT1 as pivotal “gatekeepers” of the DDR whose inhibition dismantles repair circuits and potentiates radiotherapy, offering promising avenues for combinatorial radiosensitization strategies.

#### Lysine methylation of signaling effectors in radioresponse

Beyond chromatin, lysine methylation of signaling effectors orchestrates adaptive circuits that determine radioresponse. The SET-domain methyltransferase SETD6 monomethylates the NF-κB subunit RelA/p65 at Lys310, recruiting the ankyrin-repeat methyltransferase GLP to deposit H3K9me and enforce basal repression of NF-κB targets. Ionizing radiation triggers PKCζ-mediated phosphorylation of adjacent Ser311, displacing GLP, unleashing NF-κB–driven survival and repair genes; SETD6 knockdown in MCF-7 breast cancer cells increases IR-induced apoptosis and radiosensitizes xenografts [79].

In glioma models, the E3 ligase HACE1 competitively binds and stabilizes NRF2—by blocking KEAP1 interaction and enhancing IRES-dependent translation—thereby reducing IR-induced ROS accumulation and promoting radioresistance in U87 and LN229 cells; HACE1 silencing restores ROS-mediated cell death and IR sensitivity [80].

Collectively, dynamic methylation patterns on chromatin and DDR effectors assemble a multilayered regulatory network that dictates tumor radioresponse. At the chromatin level, BAHD1-driven accumulation of H3K9me3 and H3K27me3 fosters a compacted heterochromatic state that accelerates rapid, error‐prone DSB repair and underpins resistance; pharmacologic blockade of PRC2 with GSK126 or genetic disruption of BAHD1 re‐sensitizes resistant cells. In parallel, arginine methyltransferases PRMT5 and PRMT1 methylate key DDR factors—Mxi1 and BRCA1, respectively—promoting repair complex assembly and survival; selective inhibitors such as EPZ015666 and LLY-283 reverse these modifications to dismantle repair circuits and potentiate radiotherapy. Crucially, different tumor types appear to harness distinct methylation-based mechanisms of radioresistance. In nasopharyngeal carcinoma, excessive EZH2-driven H3K27 trimethylation creates a silenced chromatin environment that confers radioresistance, a phenotype reversible by EZH2 inhibition [74]. Conversely, in NSCLC, hyperactivated PRMT5 methylates DDR targets to enhance DNA break repair, and its suppression yields profound radiosensitization [75]. These contrasts highlight that the dominant methylation “code” for radioresponse varies by cancer type, necessitating tailored targeting of either lysine methylation (e.g., EZH2/PRC2 in certain solid tumors) or arginine methylation (e.g., PRMT5 in others) to overcome radioresistance (Fig. 2D).

### Acetylation: epigenetic Yin–Yang in tumor radiosensitivity

Complementing methylation, lysine acetylation of histones and non-histone proteins exerts a yin–yang influence on chromatin dynamics and cell fate following irradiation. This PTM is catalyzed by acetyl-CoA-dependent histone acetyltransferases (HATs)—notably the MYST family member TIP60, which acetylates histone H4K16 and activates ATM to coordinate DNA repair, and the paralogs p300/CBP—and is reversed by histone deacetylases (HDAC1–3, HDAC6) and NAD⁺-dependent sirtuins [81, 82]. Beyond chromatin, acetylation of p53 at Lys120 by TIP60 and hMOF enhances its pro-apoptotic transactivation program in response to DNA damage, whereas p300/CBP-mediated acetylation at Lys382 modulates checkpoint recovery and DDR factor recruitment [83, 84]. Dysregulated acetylation dynamics underlie tumor radioresistance. In PAX3-FOXO1 fusion-positive rhabdomyosarcoma, genetic or pharmacologic inhibition of class-I HDACs (including HDAC3) prolongs γH2AX foci and induces radiosensitization in vitro and in vivo [85]. Similarly, pan-HDAC inhibitors such as vorinostat abrogate DNA repair protein loading and enhance IR-induced cell death in head and neck cancer models [86]. These findings delineate an epigenetic yin–yang: global hyperacetylation weakens chromatin compaction and impairs repair, while locus-specific acetylation of survival genes may buffer radiation stress. Rational modulation of the acetylome via HAT activators or selective HDAC inhibitors thus offers a promising strategy to tip the balance toward tumor cell killing.

#### Histone acetylation and chromatin accessibility in radiosensitivity

Ionizing radiation causes DNA breaks that must be accessed by repair enzymes. Chromatin structure, modulated by histone acetylation, is thus pivotal. Hyperacetylated chromatin (histones H3 and H4 acetylated at various lysines) is more relaxed, potentially allowing better access for repair factors but also enabling heightened expression of pro-survival genes [87, 88]. Recent analyses have demonstrated that HDAC10 overexpression in cervical cancer cells diminishes histone H3 and H4 acetylation, a change that correlates with accelerated tumor progression and the development of radioresistance [89, 90]. HDAC10 appeared to promote an aggressive phenotype by two routes: (1) deacetylating and repressing genes like *MMP2/7* and *Wnt pathway inhibitors*, thereby enhancing invasion, and (2) via a non-canonical action, upregulating TXNIP (an inhibitor of glucose uptake) through miR-223 suppression, which in turn modulates redox state and growth [91, 92]. While that study focused more on carcinogenesis than radiosensitivity, it underscores how HDAC-mediated hypoacetylation can create a pro-tumor environment. Notably, it was also observed that glioblastoma patients with high HDAC4 or HDAC6 expression had significantly worse outcomes after radiotherapy, suggesting these HDACs contribute to radioresistance [90]. Consistently, higher levels of the NuRD chromatin remodeling complex (which includes HDAC1/2) were found in radiation-insensitive rectal cancers compared to sensitive ones [93]. Together, these clinical correlations imply that excessive deacetylation (low histone acetylation) in tumors often signals a radioresistant state.

#### HATs, viral oncoproteins, and radiosensitivity

Histone acetyltransferases can also influence radioresponse through specific gene targets. In HPV-positive cancers (like cervical cancer), oncoproteins E6/E7 disable p53 and Rb, contributing to radioresistance. Acetylation has a role here: p300 (a HAT) was shown to acetylate histones H3K27, H3K9, and H4K16 at the HPV18 long control region, increasing transcription of E6/E7 oncogenes [94]. This would exacerbate radioresistance by further suppressing p53-mediated apoptosis. Conversely, another HAT, Tip60, acetylates H3K27 and H4K5 in a way that represses HPV E6/E7 expression, thus Tip60 opposed p300’s effect and acted more like a tumor suppressor in this context [95]. Although this example is specific, it highlights that acetylation of promoter chromatin can govern oncogene levels that modulate how cells respond to DNA damage. In line with this, enhancing global acetylation by HAT activation or HDAC inhibition might tilt the balance toward pro-death pathways in some settings. In breast cancer models, it was reported that increasing histone H3 acetylation combined with radiotherapy led to ROS accumulation and triggered ferroptosis, thereby overcoming radioresistance [96]. This suggests a complex scenario where the *pattern* of acetylation across the genome (which genes are up vs. down) matters more than simply “more or less” acetylation.

#### Non coding RNA–acetylation crosstalk governing radioresistance

Non-coding RNAs (ncRNAs) have emerged as precise modulators of histone acetylation at discrete genomic loci, thereby dictating tumor radioresponse. A striking example is the small nucleolar RNAs(snoRNAs) SNORA28, which is markedly overexpressed in radioresistant colorectal cancers. SNORA28 functions as a molecular decoy to recruit the bromodomain “reader” BRD4 to the promoter of LIFR, locally increasing H3K9 acetylation, upregulating LIFR transcription and activating the JAK1/STAT3 axis, ultimately promoting proliferation and radioresistance in vitro and in vivo [97]. Beyond snoRNAs, microRNAs likewise intersect with acetylation machinery under irradiation. In esophageal squamous carcinoma ECA-109 cells rendered radioresistant, ectopic expression of miR-34a downregulates the deacetylase SIRT1, resulting in accumulation of acetylated p53 and delayed γH2AX foci resolution upon IR [98]. This shift in acetylation dynamics restores apoptosis and reverses radiation resistance both in vitro and in xenograft models. Together, these studies illustrate how ncRNAs can specify both “writer” (HAT recruitment via BRD4) and “eraser” (HDAC/SIRT1 suppression) functions to sculpt the acetylome at critical repair and survival genes. Targeting such ncRNA acetylation networks through BET inhibitors or miRNA mimics offers a promising, highly selective avenue for radiosensitization.

#### HDAC inhibition strategies for radiosensitization

The central role of histone deacetylases (HDACs) in fortifying tumor radioresistance has established them as highly tractable therapeutic targets. This has spurred the clinical development of HDAC inhibitors (HDACi) as potent radiosensitizing agents, a strategy underpinned by compelling preclinical evidence. Mechanistically, HDACi function by preventing the deacetylation of both histone and non-histone proteins critical for the DDR and cell survival. For instance, in malignant meningioma, ionizing radiation (IR) induces HDAC6, which deacetylates substrates like α-tubulin and HSP90 to accelerate DDR checkpoint recovery. Pharmacological inhibition of HDAC6 not only abrogates this adaptive upregulation but also stalls DNA repair, evidenced by prolonged γH2AX foci retention, and suppresses pro-survival signaling (e.g., β-catenin/c-Myc), thereby synergizing with IR to induce robust cytotoxicity [82] (Fig. 2E).

Importantly, the balance of HAT and HDAC activity in determining radiosensitivity differs between malignancies. In cervical cancer, high expression of HDAC1 creates a deacetylated chromatin environment that activates the HIF-1α/VEGF signaling axis and significantly reduces radiotherapy sensitivity [99]. In glioblastoma, strong HDAC4 and HDAC6 expression correlates with poor clinical response to chemoradiotherapy, and genetic silencing of either enzyme radiosensitizes glioblastoma cells by impairing DNA double-strand break repair and stem-like properties [90]. By contrast, in preclinical models of breast cancer, epigenetic agents that enhance histone acetylation cooperate with radiotherapy to increase reactive oxygen species and trigger ferroptotic cell death, thereby restoring radiosensitivity in otherwise resistant tumors [100]. Thus, each tumor type exhibits a distinct “acetylation landscape” that shapes its radiation response, highlighting the need for context-specific epigenetic strategies.

### Lactylation: metabolic reprogramming meets DNA repair

Finally, bridging the gap between cellular metabolism and epigenetic regulation is protein lactylation, a recently discovered PTM that plays an increasingly recognized role in radioresponse. Lactylation was first described in 2019 as an acylation modification derived from lactate, the end-product of glycolytic metabolism. Tumor cells often exhibit the Warburg effect, characterized by high glycolytic flux even in oxygen-rich conditions, which leads to lactate accumulation in the tumor microenvironment. It is now clear that lactate is not merely a metabolic waste but can act as a signaling molecule and a substrate for lysine lactylation (Kla) on histone and non-histone proteins [101, 102]. Subsequent reviews have positioned lactylation at the nexus of metabolic reprogramming, epigenetic regulation, and therapeutic resistance [103]. Mechanistically, lactylation is installed by specific lysine lactyltransferases (“writers”) such as TIP60 for the MRN complex subunit NBS1 and CREB-binding protein (CBP) for the recombination factor MRE11, which use lactyl-CoA as the acyl donor. Conversely, class I histone deacetylases, particularly HDAC3, serve as “erasers” that remove these marks to reset chromatin states. Lactylation of NBS1 at lysine 388 stabilizes the MRE11-RAD50-NBS1 complex and promotes homologous recombination (HR) repair of DNA double-strand breaks [104]. Similarly, CBP-mediated lactylation of MRE11 at lysine 673 enhances DNA end resection and the recruitment of downstream repair factors [105]. Pharmacologic inhibition of lactate production via LDHA inhibitors or blockade of these writer enzymes reduces Kla levels, impairs HR, and sensitizes tumors to radiotherapy [106]. This emerging lactylation–de-lactylation axis thus bridges tumor metabolism and the DNA damage response, revealing targetable vulnerabilities to overcome radioresistance.

#### Lactate-driven metabolic and epigenetic reprogramming in radioresistance

Clinically, elevated intratumoral lactate levels have been demonstrated to be associated with unfavorable outcomes and radioresistance. In NSCLC, elevated LDHA expression predicts a worse prognosis. Pharmacologic inhibition of LDHA by oxamate markedly increases radiosensitivity of A549 and H1975 cells, enhancing IR-induced apoptosis, autophagy, ROS accumulation, ATP depletion, and G2/M arrest [107]. In head-and-neck cancers, regions exhibiting elevated lactate concentrations demonstrated reduced responsiveness to fractionated irradiation [108]. In orthotopic HNSCC xenografts, mean tumor lactate concentrations (7.3–25.9 µmol/g) exhibited a strong correlation with TCD50 values under a 30-fraction irradiation regimen (*R* = 0.98, *p* = 0.003), supporting lactate as a legitimate marker of radioresistance [109]. These observations establish a foundation for exploring how lactate-induced protein modifications might mechanistically drive radioresistance.

Mechanistically, Zhang et al. first uncovered histone lysine lactylation in bacterially challenged macrophages, mapping 28 Kla sites that accumulate during late-phase M1 polarization to activate homeostatic genes (e.g., ARG1) [102]. In tumors, an immunosuppressive microenvironment is often lactate-rich; accordingly, histone lactylation in tumor-associated macrophages (TAMs) promotes their polarization to an M2 phenotype, with increased ARG1, VEGF, and HIF-2α expression that supports angiogenesis and tissue repair rather than immune attack [110, 111]. This suggests that lactylation in the TME can dampen anti-tumor immunity (discussed further in a later section), indirectly aiding tumor radioresistance by blunting immune-mediated tumor control. Beyond chromatin, extracellular lactate engages GPR81 on tumor cells to inhibit cAMP/PKA and activate YAP/TAZ, thereby inducing PD-L1 expression and impairing CD8⁺ T-cell function [112, 113]. Thus, a high-lactate environment both modifies proteins and activates pathways that allow tumors to better withstand radiotherapy, through metabolic flexibility and immune evasion.

#### Lactylation of DNA repair proteins as determinants of radiosensitivity

Breakthrough studies have revealed that Kla directly modifies key DNA repair factors, accelerating repair and promoting resistance to genotoxic therapies. Guanzhang Li et al. demonstrated that in glioblastoma stem cells with high ALDH1A3 expression, ALDH1A3-driven PKM2 tetramerization elevates intracellular lactate and drives site-specific lactylation of XRCC1 at K247. Lactylated XRCC1 exhibits increased importin-α binding, enhances nuclear translocation, and accelerates single‐strand break repair, conferring chemoradiotherapy resistance; disruption of the ALDH1A3-PKM2 interaction by small‐molecule D34-919 prevents XRCC1 lactylation and restores radiosensitivity both in vitro and in xenograft models [114]. This work not only identifies XRCC1 lactylation as a new mechanism of therapeutic resistance, but also suggests a tangible strategy to counter it by targeting tumor metabolism.

Recent work by Yu and colleagues has broadened our understanding of protein lactylation in DNA repair, identifying NBS1 and histone H2B as novel lactylation targets and demonstrating that lysine lactylation modulates homologous recombination efficiency and checkpoint activation across multiple tumor models, while also proposing Kla-based biomarkers for real-time monitoring of therapeutic response [103]. For example, if histones near DNA break sites become lactylated, it could alter chromatin accessibility or the binding of repair complexes. Indeed, lactate was shown to induce hyperacetylation of histones H3 and H4 by inhibiting histone deacetylases (HDACs) in cervical cancer cells [115]; by analogy, histone lactylation might also modulate chromatin in ways that favor pro-survival gene expression under radiation stress. The review notes a *paradoxical nature* of lactylation: on one hand, increased lactylation can promote *immune suppression and radioresistance*; on the other, blocking lactylation pathways could sensitize tumors to radiotherapy and even improve immunotherapy outcomes [103].

Complementing these findings, studies in cervical carcinoma cells show that exogenous L- and D-lactate enter nuclei via MCT1 and MCT4 and inhibit class I and II HDACs, inducing hyperacetylation of histones H3 and H4. This chromatin relaxation promotes recruitment of NHEJ and HR effectors, including DNA-PKcs, BRCA1, and NBS1, to DNA lesions, a mechanism that parallels lactylation-driven repair enhancement [115].

#### Therapeutic targeting of lactylation for radiosensitization

Targeting lactylation-dependent repair emerges as a novel metabolic-epigenetic strategy for radiosensitization. In glioblastomas with high ALDH1A3 expression, D34-919 effectively disrupts PKM2 tetramerization, reduces XRCC1 lactylation, and synergizes with chemoradiotherapy in patient-derived xenografts [114]. In gastric cancer models, clinical LDHA inhibitor stiripentol lowers lactate levels, abolishes NBS1 K388 lactylation, impairs MRN complex assembly and homologous recombination, and markedly enhances cisplatin and IR efficacy in PDX mice [104]. Beyond enzyme inhibitors, blockade of lactate transport (e.g., MCT1/4 inhibitors) or antagonism of lactate receptor HCAR1 offers additional avenues to limit nuclear lactate influx and downstream Kla [103]. Looking forward, ongoing research aims to validate lactylation signatures as biomarkers to stratify patients for metabolically targeted radiosensitization interventions. As with other PTMs, the impact of lactylation on radioresponse varies by tumor context. In glycolytic tumors such as glioblastoma, ALDH1A3-driven lactate accumulation promotes direct lactylation of DNA repair proteins such as XRCC1, markedly accelerates repair kinetics and confers resistance to chemoradiotherapy [114]. In head-and-neck cancers, elevated lactate levels in the TME induce histone lactylation programs in tumor and myeloid compartments, promoting immunosuppressive phenotypes including M2-like TAM polarization and CD8⁺ T cell dysfunction, which indirectly undermines radiosensitivity [116, 117]. These distinct mechanisms, one predominantly cell intrinsic and the other largely microenvironment mediated, highlight the tumor specific ways in which lactate and lactylation influence radiotherapy outcomes (Fig. 2F).

## Therapeutic targeting of PTMs to overcome radioresistance

Post-irradiation PTM networks regulate DNA repair, cell-death pathways, and immune evasion, offering multiple therapeutic entry points. Selective inhibition of PTM “writers,” “erasers,” or “readers” can undermine tumor cell repair capacity, shift the balance toward apoptosis or ferroptosis, and remodel the TME to enhance anti-tumor immunity.

### Targeting DNA repair and survival pathways

Clinical proof-of-concept for PTM-targeted radiosensitizers has been established with PARP inhibitors. In a Phase I/II trial (NCT01236560), vorinostat (400 mg daily on days 1–7 of a 28-day cycle) combined with radiotherapy ± temozolomide was well tolerated in newly diagnosed glioblastoma patients, with no grade ≥ 3 late toxicities observed [118]. Preclinically, olaparib plus radiotherapy activated STING-dependent type I interferon signaling and synergized with anti–PD-L1 in pancreatic ductal adenocarcinoma models, achieving a 33% complete response rate [119]. Selective HDAC6 inhibition has also shown promise: in meningioma models, ionizing radiation upregulates HDAC6, and pre-treatment with the HDAC6-selective inhibitor Cay10603 prolonged γH2AX foci persistence by 48 h and increased IR-induced cytotoxicity by 2-fold [82]. PRMT5 inhibitors such as LLY-283 prevent symmetric dimethylation of repair effectors (e.g., Mxi1), stall homologous recombination, and sensitize U251 glioma and PSN1 pancreatic xenografts to radiotherapy, reducing tumor volume by 60% versus RT alone [71]. SUMO pathway blockade with the E1 inhibitor TAK-981 disrupts SUMOylation of MDC1 and RNF168, impairs DNA double-strand break focus formation, and synergizes with RT in pancreatic and hematologic malignancy models, prolonging median survival from 28 to 45 days [120]. Finally, the deubiquitinase inhibitor VLX1570 targets USP14, induces accumulation of polyubiquitinated substrates, exacerbates IR-induced DNA damage, and drives apoptosis in multiple myeloma xenografts; however, clinical development was halted due to pulmonary toxicity observed in a Phase I trial [121]. See Table 1 for an overview of representative PTM-targeted agents evaluated as radiosensitizers in various cancers.

**Table 1:** Major PTM-Targeting inhibitors for radiosensitization (Representative Examples) and their development status (see text for details)

PTM Category	Drug (Inhibitor)	Target Enzyme/Process	Tumor Types Studied	Clinical Stage	Remarks
ADP-ribosylation	PARP inhibitors (e.g., Olaparib, Talazoparib)	PARP1/2 (poly(ADP-ribose) polymerases)	Breast (TNBC), ovarian, glioblastoma, HNSCC, others	Approved (multiple); Phase I/II trials with RT	PARP1/2 inhibition & trapping enhances DNA damage; effective in HR-deficient tumors; clinical combos with RT show tolerability [122].
Acetylation	HDAC inhibitors (e.g., Vorinostat, Romidepsin)	Histone deacetylases (pan-HDAC or class-selective)	Glioblastoma, head & neck, T-cell lymphoma, others	Approved (some subtypes); Phase I/II with RT	Induce hyperacetylation, disrupt DNA repair and survival signaling; vorinostat + RT well tolerated in GBM trial [118].
Methylation	PRMT5 inhibitors (e.g., LLY-283, JNJ-64619178)	Protein arginine methyltransferase 5	Glioma, pancreatic, NSCLC, breast (preclinical)	Preclinical; Phase I ongoing	Block symmetric arginine methylation, impair HR repair; LLY-283 sensitized glioma & pancreatic xenografts to RT [71].
SUMOylation	SUMO E1 inhibitor (TAK-981, subasumstat)	SUMO-activating enzyme (SAE1/UBA2)	Pancreatic, lymphoma, AML (preclinical); solid tumors, lymphomas (clinical)	Phase I/II ongoing	Inhibits global SUMO conjugation, prolongs DNA damage signaling; enhances RT efficacy in models [117]; emerging immunomodulatory effects (IFN induction) [123].
Neddylation	NAE inhibitor (MLN4924, pevonedistat)	NEDD8-activating enzyme (NAE1/UBA3)	AML/MDS (Phase II); solid tumors: HNSCC, pancreatic, prostate, breast (preclinical)	Phase I/II (multiple trials)	Inactivates cullin-RING ligases, causes accumulation of cell-cycle inhibitors (p21, WEE1, CDT1); potent radiosensitizer across models [58, 124]; combinatorial eval. with chemo/RT and immunotherapy underway.

### Reprogramming the tumor microenvironment and immune response

**Fig. 3:**
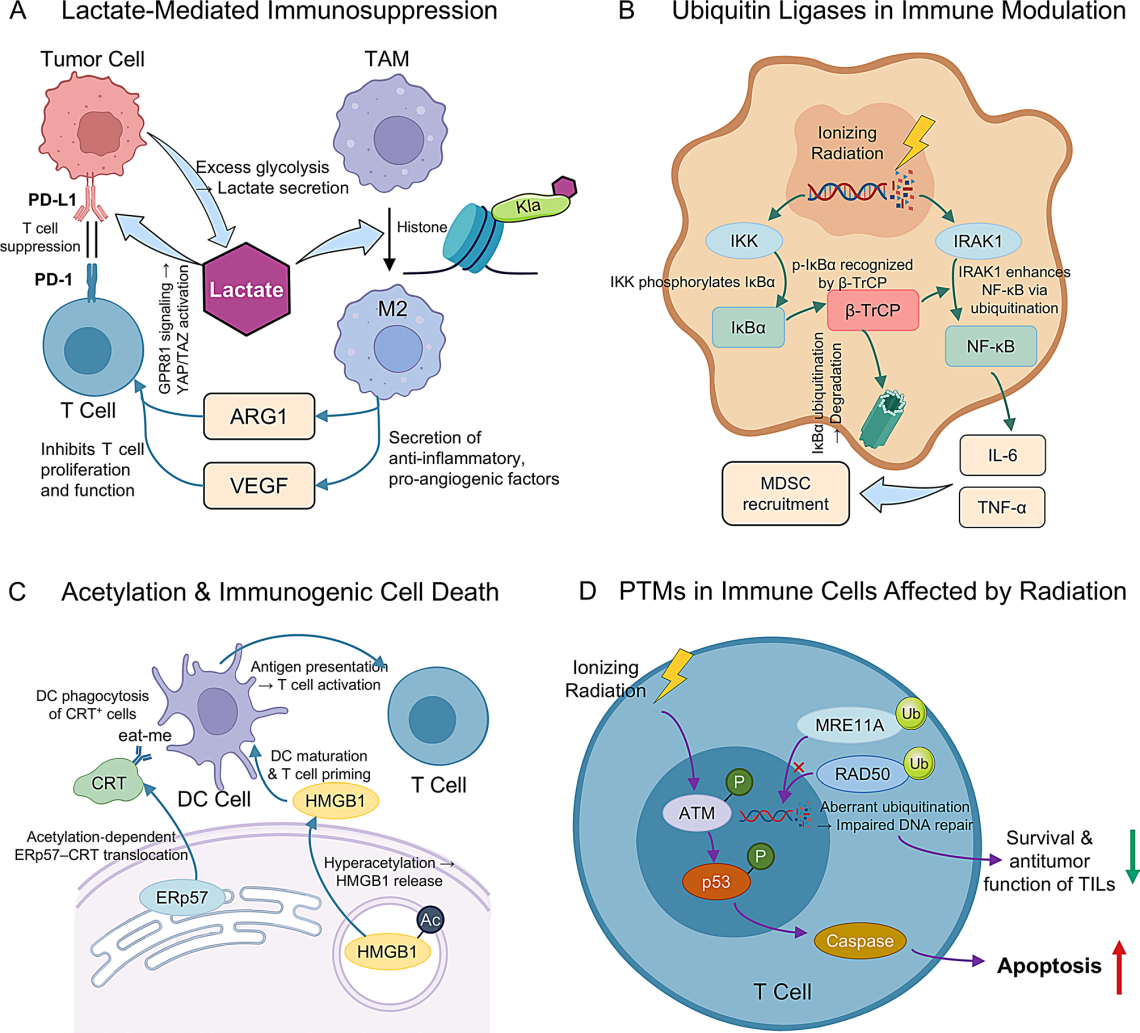
Immune-microenvironment modulation by PTMs and implications for radiotherapy. (**A**) Lactate-mediated immunosuppression. Tumor-derived lactate induces histone lactylation in macrophages, promoting an M2-like phenotype (ARG1/VEGF) and, via GPR81 signaling, increases tumor PD-L1 expression that suppresses T-cell function. (**B**) Ubiquitin ligases in immune modulation. After IR, IKK-driven phosphorylation and β-TrCP-mediated ubiquitination degrade IκBα, activating NF-κB and pro-inflammatory cytokines (IL-6, TNF-α) that recruit MDSCs; IRAK1 further enhances NF-κB signaling via ubiquitination pathways. (**C**) Acetylation and immunogenic cell death (ICD). ER-stress induced CRT–ERp57 surface translocation (“eat-me” signal) enables dendritic-cell phagocytosis; HMGB1 hyperacetylation promotes its extracellular release to support antigen presentation and T-cell priming. (**D**) PTMs within irradiated T cells. ATM-p53 signaling drives apoptosis, while aberrant ubiquitination of MRN components (MRE11/RAD50) can impair repair and diminish TIL survival. Overall, PTM rewiring shapes antitumor immunity after RT and provides combinatorial targets with checkpoint blockade. CRT, calreticulin; DC, dendritic cell; HMGB1, high-mobility group box 1; ICD, immunogenic cell death; IKK, IκB kinase; MDSC, myeloid-derived suppressor cell; TIL, tumor-infiltrating lymphocyte. Schematic; not to scale

PTM-targeted radiosensitization can be complemented by reprogramming lactate-driven immunosuppressive circuits in the TME. Tumor-derived lactate fosters an immunosuppressive milieu by inducing histone lactylation in TAMs, which drives their polarization toward an M2-like, pro-tumor phenotype; conversely, genetic ablation of PERK in myeloid cells abrogates this lactylation, restores CD8⁺ T-cell infiltration, and delays glioblastoma progression [125, 126]. Inhibition of LDHA with oxamate reduces intratumoral lactate levels, repolarizes TAMs toward a pro-inflammatory M1 state, and restores effector CD8⁺ T-cell proliferation in murine Lewis lung carcinoma models [127]. Pharmacologic blockade of lactate export via the MCT1 inhibitor AZD3965 diminishes histone lactylation in tumor-associated myeloid cells, enhances dendritic-cell and natural-killer-cell infiltration, and synergizes with PD-1 blockade to delay lymphoma xenograft growth [128]. Finally, targeting the lactate receptor HCAR1 with reserpine impairs recruitment of immunosuppressive CCR2⁺ PMN-MDSCs, augments CD8⁺ T-cell–dependent antitumor immunity, and sensitizes colorectal tumors to PD-1 blockade [129]. Together, these studies support the integration of PTM-targeted radiosensitizers with lactate-signaling modulators to convert “cold” tumors into immunologically “hot” ones and thereby enhance the efficacy of combined radio-immunotherapy (Fig. 3A).

### Ubiquitin-mediated pathways in immune cells

Ionizing radiation markedly induces IRAK1 expression in glioma via a STING–FOXA2 axis; IRAK1 phosphorylates Beclin-1 to initiate pro-survival autophagy and activates canonical NF-κB, thereby driving radioresistance. Genetic silencing or pharmacologic inhibition of IRAK1 abrogates autophagy, enforces G₂/M arrest, enhances caspase-3–mediated apoptosis, and restores RT sensitivity in orthotopic glioma xenografts [37]. Within the TME, the E3 ligase TRAF6 is upregulated in tumor-infiltrating MDSCs, where K63-linked ubiquitination of STAT3 by TRAF6 sustains STAT3 phosphorylation, arginase-1 expression, and suppressive MDSC activity; TRAF6 knockdown mitigates MDSC-mediated T-cell inhibition and synergizes with RT to enhance antitumor immunity in murine carcinoma models [130]. Finally, proteasome inhibition with bortezomib stabilizes IκBα, suppresses NF-κB–driven pro-survival cytokine release, and synergizes with RT to potentiate apoptosis in head-and-neck cancer xenografts [131]. Collectively, these studies validate ubiquitin E3 ligases and the proteasome as viable targets to rewire radiation-induced immunosuppression and bolster radiotherapeutic efficacy (Fig. 3B).

### Promoting Immunogenic cell death (ICD) via PTM modulation

PTM modulators can amplify RT-induced ICD by enhancing DAMP exposure and release. Surface translocation of calreticulin (CRT), the pre‐apoptotic “eat‐me” signal, requires ERp57 co‐translocation under ER‐stress induced by irradiation; this CRT–ERp57 complex is essential for dendritic‐cell phagocytosis and tumor‐specific CTL priming [132, 133]. Concurrently, inhibition of class I histone deacetylases with vorinostat or entinostat hyperacetylates HMGB1, facilitating its active secretion from dying tumor cells; extracellular HMGB1 engages TLR4 on dendritic cells to promote antigen cross‐presentation, and in ESCC patients undergoing chemoradiotherapy, elevated serum HMGB1 correlates with improved three‐year overall survival and increased intratumoral CD8⁺ TIL density [134, 135]. Additionally, PTMs of the autophagy machinery, such as LAMP1-dependent lysosomal ATP packaging, coordinate extracellular ATP release during ICD; ATP then acts as a “find‐me” signal to recruit and activate antigen‐presenting cells, and genetic or pharmacologic enhancement of autophagy augments ATP secretion and synergizes with RT to potentiate antitumor immunity in murine colorectal carcinoma models [136, 137]. Together, these findings highlight how targeting PTMs of ER‐stress sensors, deacetylases, and autophagy regulators can strengthen ICD and improve the efficacy of combined radio-immunotherapeutic regimens (Fig. 3C).

### Integrating PTM modulators with radio-immunotherapy

Recent preclinical evidence indicates that strategic combinations of PTM-targeted agents with radiotherapy (RT) and immune checkpoint blockade (ICB) can produce profound synergistic antitumor effects. In pancreatic ductal adenocarcinoma models, olaparib plus fractionated RT induces a cGAS–STING–IFN type I response that is further amplified by anti–PD-L1 therapy, resulting in marked increases in intratumoral CD8⁺ T-cell infiltration and durable tumor control [119, 138]. Class I-selective HDAC inhibitors have also proven effective radiosensitizers and immunomodulators. OKI-179 enhances histone acetylation at interferon-responsive promoters, upregulates MHC-I expression, and when combined with RT and PD-1 blockade extends survival in murine breast and lung carcinoma models [139, 140]. Nanoparticle (NP)-based delivery platforms offer tumor-restricted release of PTM modulators and co-stimulatory agents. For example, dual-function A2-CL/Dbait NPs and A2-PLGA-PEG/anti-OX40 NPs co-delivered with RT potentiate OX40-mediated T-cell co-stimulation and elicit robust abscopal responses in GBM xenografts [141]. Similarly, NP-enhanced RT plus anti–PD-L1 limits post-surgical recurrence and metastasis in breast cancer models [142].

Future directions will deploy isoform-selective PRMT inhibitors to minimize normal-tissue toxicity, refine NP carriers for spatiotemporal control of PTM activity, and optimize sequencing of RT and ICB. High-plex spatial proteomics techniques (e.g., imaging mass cytometry and MALDI-MSI) now enable in situ mapping of PTM “hotspots” within irradiated tumors, guiding rational design of next-generation combinatorial regimens that dismantle DNA-repair, prosurvival, and immunosuppressive circuits in concert [143] (Fig. 3D).

In summary, the immunological outcomes of radiotherapy are intricately governed by dynamic PTM networks that orchestrate both “find-me” and “kill-me” signals. A truly integrative strategy must therefore extend beyond the direct inhibition of DNA repair to encompass the modulation of radiation-induced immunosuppressive PTMs. Clinical and preclinical data already support the combination of radiotherapy with immune checkpoint blockade, and the addition of PTM-targeted agents—such as lactate transport inhibitors or PARP7 antagonists—promises to further amplify antitumor immunity. Looking ahead, mapping the spatial and temporal “ubiquitin landscape” of immune checkpoints and decoding the “acetylation code” of cytokine loci in irradiated tumors will be critical. By leveraging these insights, we can design next-generation regimens that simultaneously dismantle DNA-repair machineries, disable prosurvival signaling, and unmask tumors to immune attack, ultimately realizing the full potential of combined radio-immunotherapy.

## Conclusion and future perspectives

The interplay between post-translational modifications and radiation response has shifted from a peripheral concept to a central paradigm in radiobiology. An expanding repertoire of modifications—ADP ribosylation, ubiquitination, neddylation, SUMOylation, methylation, acetylation and lactylation—regulates DNA damage sensing, repair pathway choice and cell fate after irradiation.

Decoding the hierarchical crosstalk among these modifications will require integrated proteomic mapping that captures temporal dynamics and site specificity. Such efforts promise to reveal how early bursts of PARylation prime ubiquitin signalling or how acetylation influences subsequent methylation patterns. Parallel advances in PTM targeted imaging and mass spectrometry will enable biomarker development for personalized radiotherapy by identifying PTM signatures that predict tumor response or resistance. Clinically, combining radiotherapy with modulators of PTM enzymes offers a means to sensitize tumors while sparing normal tissue: initial trials of PARP inhibitors combined with radiation, or of HDAC inhibitors combined with radiation, have demonstrated safety and suggested improved tumor control. Future strategies will extend this approach to lactate metabolism inhibitors and specific PARP isoform antagonists such as PARP7 and optimize scheduling and tumor-selective delivery via nanoparticle carriers to enhance efficacy and minimize toxicity. Co targeting PTMs and non-coding RNA pathways promises a dual layer of intervention capable of collapsing tumor survival networks more effectively. Finally, integrating PTM modulation with immunotherapy can reprogram the tumor microenvironment toward sustained antitumor immunity: combinations of radiation, immune checkpoint blockade and PTM targeted agents have the potential to convert immunologically cold tumors into hot tumors that engage durable immune responses. By translating these molecular insights into precision combination regimens, the field of radiotherapy is poised for a transformative era in which treatment-resistant malignancies become curable through rationally designed multi modal therapies.

## Data Availability

No datasets were generated or analysed during the current study.

## Abbreviations

ARH3: ADP-ribosylhydrolase 3
BER: Base Excision Repair
DDR: DNA Damage Response
DSBs: Double-strand breaks
EZH2: Enhancer of zeste homolog 2
GSC: Glioblastoma stem-like cells
HAT: Histone acetyltransferase
HDAC: Histone deacetylase
HDACi: Histone deacetylase inhibitor
HNSCC: Head-and-neck squamous cell carcinoma
HR: Homologous recombination
IR: Ionizing radiation
lncRNA: Long non-coding RNA
MAR: Mono-ADP-ribosylation
ncRNA: Non-coding RNA
NHEJ: Non-homologous end joining
PAR: Poly(ADP-ribose)
PARG: Poly(ADP-ribose) glycohydrolase
PARP: Poly(ADP-ribose) polymerase
PRMT: Protein arginine methyltransferase
PTM: Post-translational modification
SAM: S-adenosylmethionine
snoRNA: Small nucleolar RNA
SSB: Single-strand break
TME: Tumor microenvironment
TNBC: Triple-negative breast cancer
